# Investigation of a foodborne outbreak at a mass gathering in Petaling District, Selangor, Malaysia

**DOI:** 10.5365/wpsar.2022.13.1.860

**Published:** 2022-02-10

**Authors:** Sudeash Rajakrishnan, Muhd Zulfadli Hafiz Ismail, Syed Hafeez Jamalulail, Norazmalia Alias, Hassan Ismail, Salina Md Taib, Lee Soo Cheng, Zazarida Zakiman, Ong Richai, Rubaan Raj Silverdurai, Mohamad Paid Yusof

**Affiliations:** a District Health Office of Petaling, Selangor, Ministry of Health Malaysia.

## Abstract

**Objective:**

On 6 October 2019, Petaling District Health Office received notification of a possible foodborne outbreak involving a mass gathering event. This report presents the processes of diagnosis verification, case identification, determination of associated risk factors and commencement of control measures in managing the outbreak.

**Methods:**

Cases were defined as those who attended the mass gathering event on 6 October 2019, consumed the pre-packaged food and subsequently developed vomiting, abdominal pain, diarrhoea or other symptoms (e.g. fever, nausea and dizziness). Epidemiological, environmental and laboratory investigations were performed. Data were analysed using SPSS software (version 24.0).

**Results:**

A total of 169 cases were identified. The attack rate was 7.2%, and cases ranged in age from 7 to 50 years, with a median of 20 years. A total of 156 (92.3%) cases had vomiting, 137 (81.1%) had abdominal pain and 83 (49.1%) had diarrhoea. Consuming nasi lemak at the mass gathering was found to be significantly associated with developing illness (odds ratio: 9.90, 95% confidence interval: 6.46–15.16). The samples from suspected food, food handlers and the environment were positive for *Bacillus cereus*, *Staphylococcus aureus* or coliforms.

**Discussion:**

The outbreak at this mass gathering was probably caused by food contaminated with *B. cereus* and *S. aureus*. To prevent future outbreaks, we recommend mass gathering events use certified catering services that have adequate food safety training.

An outbreak of food poisoning occurred during a mass gathering held on 6 October 2019 in Petaling District, Selangor, Malaysia. The event was attended by about 20 000 people (politicians, members of the public and students from four public universities). At the event, pre-packaged food was provided by two caterers. The Petaling District Health Office received an initial notification of food poisoning involving attendees of the event from a hospital in Klang District. This report describes the outbreak investigation to identify cases, contaminated food items and the causative pathogen(s), to determine associated risk factors and to describe the prevention and control measures taken by the District Health Office to manage the outbreak.

## Methods

### Epidemiological investigation

The case definition was any person who attended the mass gathering event in Petaling District, consumed the pre-packaged food and subsequently developed abdominal pain, diarrhoea, vomiting or other constitutional symptoms (e.g. fever, nausea or dizziness) on 6 October 2019.

Active case finding was conducted by interviewing attendees of the mass gathering on site. Passive case finding was encouraged by alerting all health facilities to report cases related to the outbreak. All cases identified were interviewed using a standardized food poisoning questionnaire. Data on sociodemographic characteristics, symptoms of food poisoning, onset of symptoms and food intake history during the event were gathered.

### Environmental investigation

An environmental investigation was conducted at the two kitchen facilities involved in preparing the pre-packaged food for the mass gathering. The parameters inspected were environmental hygiene, flow of food preparation including ingredients and their sources, handling of leftover foods and equipment hygiene.

### Laboratory investigation

Rectal swabs from cases were taken at random, and environmental samples were collected focusing on suspected food and equipment used during its preparation. Clinical samples were collected in labelled containers with Cary-Blair medium. Vomitus samples were collected in sterile containers. A 250 g sample of each leftover item of food or drink (nasi lemak, bread, rice with chicken, and bottled water) was collected in sterilized sampling bags. Swab samples of food handlers’ hands and kitchen utensils (cutting boards, knives, rice cookers and food containers) were collected using 3M quick swabs. All samples were sent to the National Public Health Laboratory within 24 hours of collection for culturing and sensitivity testing.

### Analytical epidemiological investigation

An unmatched case–control study was conducted to identify and confirm the food items and risk factors contributing to the outbreak. Convenience sampling at a ratio of 1:0.3 was used, with 169 cases and 47 controls enrolled in the study. A case was defined as described above, while a control was defined as any asymptomatic individual who ate the food items provided during the event. Probability values were obtained using Fisher’s exact test.

### Data management

Respondents’ identities were kept confidential. All data were collected, collated, verified and analysed using SPSS software (version 24.0).

## Results

### Epidemiological results

Of the 2341 participants who were at risk of consuming the affected food, 169 were identified as cases for an attack rate of 7.2%. Cases’ ages ranged from 7 to 50 years, with a median of 20 years. Of the total cases, 161 were detected through active case finding and eight through passive case finding. A total of 156 (92.3%) cases reported vomiting, 137 (81.1%) had abdominal pain, 83 (49.1%) had diarrhoea and 19 (11.2%) had fever (**Table 1**). All cases sought treatment; 20 were admitted to hospital, and the remaining cases were treated as outpatients. No deaths were reported.

**Table 1:** Characteristics and symptoms of cases (*n* = 169) and controls (*n* = 47)

-	Cases, *n*(%)	Controls, *n*(%)
Sex		
Male	25 (14.8)	3 (6.4)
Female	144 (85.2)	44 (93.6)
Age group (years)		
£ 15	2 (1.2)	0 (0)
16–25	163 (96.4)	47 (100)
26–35	1 (0.6)	0 (0)
36–45	2 (1.2)	0 (0)
46–55	1 (0.6)	0 (0)
Median age (range)	20 (7–50)	21 (19–23)
Affiliation of cases		
University A students	161 (95.3)	47 (100)
General public	6 (3.6)	0 (0)
University B students	2 (1.2)	0 (0)
Symptoms		
Vomiting	156 (92.3)	0 (0)
Abdominal pain	137 (81.1)	0 (0)
Diarrhoea	83 (49.1)	0 (0)
Fever	19 (11.2)	0 (0)

**Fig. 1:**
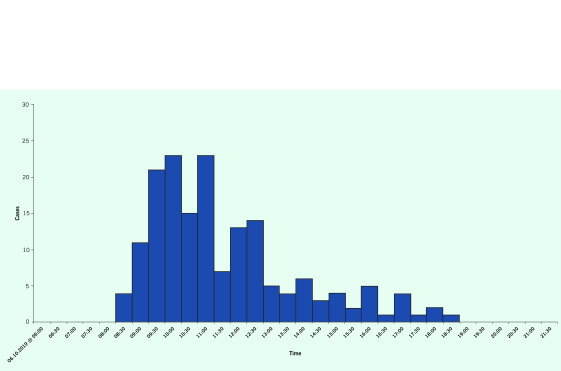
Epidemic curve of the foodborne outbreak at a mass gathering in Petaling District, Selangor on 6 October 2019

The epidemic curve suggested a point-source outbreak, with a pattern showing a rapid increase, single peak and tapered decline in the number of cases (**Fig. 1**). Exposure time was about 07:00 on 6 October 2019. The onset of the index case was at 08:30, and the last case was at 18:30. The incubation period ranged from 1.5 to 11.5 hours, with a median of 6.5 hours.

Nasi lemak had the highest attack rate, at 89.6% (*n* = 169). The odds of cases having consumed nasi lemak (odds ratio [OR]: 9.90, 95% confidence interval [CI]: 6.46–15.16) at the mass gathering were significantly higher than the odds of cases having consumed bread (OR: 0.024, 95% CI: 0.010–0.059) or rice with chicken (OR: 0.19, 95% CI: 0.14–0.27). Bottled water also had a high attack rate, at 80.8% (*n* = 160), but this was probably due to the high overlap of cases who consumed both nasi lemak and bottled water.

[**insert** Figure 1]

### Environmental examination

The overall cleanliness rating of facility A was 48.8%. Hazard analysis found poor general cleanliness and hygiene, evidenced by a congested food preparation area and dirty flooring, food preparation surfaces and sink floor. Unsuitable, defective and dirty equipment, such as a dirty chiller with a suboptimal temperature (10 °C), were used during food preparation for the mass gathering. Additionally, the components of the nasi lemak, which included rice, sambal and boiled egg, were cooked using the same pot.

Six food handlers worked at facility A. None had attended a food handling training course or had been vaccinated for typhoid. None had any acute gastroenteritis symptoms at the time of inspection. The preparation of the nasi lemak started 26 hours before consumption and the estimated holding time was up to 20 hours at room temperature.

The overall general cleanliness and hygiene of facility B were satisfactory, with a rating of 86%.

Several control measures were taken to contain and eliminate the outbreak. The caterers’ activities at the facilities were suspended temporarily under the legal provisions of Section 18(1)(d) Infectious Disease Control Act 1988, Malaysia (CDC Act 1988: Act 342). This was to ensure that the caterers could take measures to comply with the requirements outlined under the Food and Hygiene Regulation and prevent further outbreaks from occurring before resuming their activities. The caterers and food handlers also underwent food handling and health education, and they were urged to attend a food handling training course and obtain typhoid vaccinations. The organizer of the mass gathering was advised to liaise with the local health district authority before organizing future events, to ensure that preventive measures were taken, including inspection and sampling of food handling facilities.

### Laboratory results

A total of 77 samples were taken. None of the 33 rectal swabs or the nine vomitus samples tested positive for microorganisms. All five food samples were positive for at least one microorganism, namely *Bacillus cereus, Staphylococcus aureus* or coliforms. Ten of the 22 environmental samples, and three of the eight hand swabs from the food handlers, tested positive for *B. cereus* and coliforms.

## Discussion

The outbreak of food poisoning at this mass gathering was probably caused by *B. cereus* and *S. aureus*. This was consistent with the symptoms and incubation period reported, and was further supported by the laboratory results from samples of suspected foods, kitchen equipment and food handlers, which isolated *B. cereus* and *S. aureus*. The combination of the ubiquitous nature of these organisms and their coexistence in food and the environment is a major concern for food safety. Additionally, *S. aureus* food poisoning resembles that of *B. cereus* in terms of symptoms and incubation period. (*1*, *2*) Also, synergism has been observed between *S. aureus* sphingomyelinase and *B. cereus* phosphatidylcholine hydrolase. (*2*)

The most likely vehicle of the outbreak was the nasi lemak. The odds of its consumption in cases was 9.90 times that of controls, and this result was statistically significant. The other food items, despite being statistically significant, yielded odds of less than 1 (bread and rice with chicken), and the likely causative organisms are not commonly found in bottled water. *B. cereus* is a Gram-positive rod that grows well in both aerobic and anaerobic environments. It produces an emetic toxin leading to the symptoms seen in these cases. (*3*, *4*) Plant-based foods – particularly rice, pasta and noodles – are common reservoirs for *B. cereus*. (*5*, *6*) Rice is the main ingredient in nasi lemak, enhancing the likelihood of food poisoning by *B. cereus*. The laboratory findings also strengthened the evidence for B. cereus as the causative pathogen because the pathogen was found in samples from the food handlers, food and environment. Low bacterial load could explain the negative clinical samples and the mild symptoms exhibited by the cases.

*S. aureus* is a Gram-positive, sphere-shaped bacteria that is part of the normal flora of human skin and mucous membranes. (*7*) Poor hygiene practices by food handlers can increase the possibility of transferring  *S. aureus* to prepared food, (*7*) causing it to release enterotoxins that lead to the symptoms seen in this report. (*8*) The possibility of *S. aureus* causing the foodborne outbreak was further strengthened by its presence in the tested sample of nasi lemak. Additionally, the prolonged holding time of more than 4 hours increased the multiplication of the microorganisms leading to food poisoning. (*8*) Poor food safety and hygiene practices have been established as a major factor in foodborne outbreaks in Malaysia and globally. (*9*, *10*)

Regarding limitations, since food was only served after the mass gathering event was over, most participants had left the event site before investigation of the foodborne outbreak began. Also, there was no official registry of event participants. This made it difficult to identify and interview participants, and explains the low number of participants in the study and the use of convenience sampling to identify controls.

## Conclusion

This report highlights the processes undertaken to identify cases and causative pathogens of a foodborne outbreak at a mass gathering, and measures taken to avoid future outbreaks. This study showed that the outbreak was preventable. Recommendations to prevent future outbreaks include that organizers of mass gatherings engage with certified catering services only (e.g. by using the Trust MyCatering initiative established by the Ministry of Health Malaysia, which provides certification to catering operators that comply with food safety requirements); (*11*) that awareness campaigns, guidelines and policies be established to ensure that organizers of mass gatherings liaise with local health authorities to improve food handling and hygiene practices before events are held; and that stricter enforcement be considered for caterers and organizers that breach food handling and hygiene practices causing foodborne outbreaks.

## References

[1] Elhabibi T, Attia AS, Hashem A, Ashour MS. Characterization of Bacillus cereus and Staphylococcus aureus strains isolated from food and food products retailed in the Egyptian Markets. New Egypt J Microbiol. 2012;31:40–52.

[2] Kumar TD, Murali HS, Batra HV. Simultaneous detection of pathogenic B. cereus, S. aureus and L. monocytogenes by multiplex PCR. Indian J Microbiol. 2009 Sep;49(3):283–9. 10.1007/s12088-009-0032-y 23100783 PMC3450020

[3] Dietrich R, Jessberger N, Ehling-Schulz M, Märtlbauer E, Granum PE. The food poisoning toxins of Bacillus cereus. Toxins (Basel). 2021 01 28;13(2):98. 10.3390/toxins13020098 33525722 PMC7911051

[4] Granum PE, Lund T. Bacillus cereus and its food poisoning toxins. FEMS Microbiol Lett. 1997 Dec 15;157(2):223–8. 10.1111/j.1574-6968.1997.tb12776.x 9435100

[5] Agata N, Ohta M, Yokoyama K. Production of Bacillus cereus emetic toxin (cereulide) in various foods. Int J Food Microbiol. 2002 Feb 25;73(1):23–7. 10.1016/S0168-1605(01)00692-4 11883672

[6] Kramer JM, Gilbert RJ. Bacillus cereus and other Bacillus species. In: Doyle MP, editor. Foodborne bacterial pathogens. New York: Marcel Dekker, Inc.; 1989. pp. 21–70.

[7] Hennekinne JA, De Buyser ML, Dragacci S. Staphylococcus aureus and its food poisoning toxins: characterization and outbreak investigation. FEMS Microbiol Rev. 2012 Jul;36(4):815–36. 10.1111/j.1574-6976.2011.00311.x 22091892

[8] Hennekinne JA. Staphylococcus aureus as a leading cause of foodborne outbreaks worldwide. In: Fetsch A, editor. Staphylococcus aureus. London: London Academic Press; 2018. pp. 129–46. 10.1016/B978-0-12-809671-0.00007-3

[9] Lema K, Abuhay N, Kindie W, Dagne H, Guadu T. Food hygiene practice and its determinants among food handlers at University of Gondar, Northwest Ethiopia, 2019. Int J Gen Med. 2020 11 16;13:1129–37. 10.2147/IJGM.S262767 33235486 PMC7679353

[10] Xu H, Zhang W, Guo C, Xiong H, Chen X, Jiao X, et al. Prevalence, serotypes, and antimicrobial resistance profiles among Salmonella isolated from food catering workers in Nantong, China. Foodborne Pathog Dis. 2019 05;16(5):346–51. 10.1089/fpd.2018.2584 30657345

[11] Food Safety and Quality Division. Trust MyCatering. Federal Territory of Putrajaya: Ministry of Health Malaysia; 2021. Available from: http://fsq.moh.gov.my/v6/xs/page.php?id=12, accessed 4 November 2021.

